# Beta-lactam comprehensive allergy management program in a community medical center

**DOI:** 10.1017/ash.2023.461

**Published:** 2023-10-27

**Authors:** Lakhini Vyas, Karan Raja, Susan Morrison, Donald Beggs, Mark S. Attalla, Mitesh Patel, Mona Philips

**Affiliations:** 1 Pharmacy Department, Clara Maass Medical Center, Belleville, NJ, USA; 2 Ernest Mario School of Pharmacy, Rutgers University, New Brunswick, NJ, USA; 3 Department of Medicine, Clara Maass Medical Center, Belleville, NJ, USA

## Abstract

**Objective:**

The Beta-lactam Comprehensive Allergy Management Program (CAMP) was implemented to facilitate complete beta-lactam allergy history documentation in the electronic medical record (EMR) and increase beta-lactam utilization. The study objective was to assess the rate of complete allergy histories and days of antimicrobial therapy (DOT) before versus after CAMP implementation.

**Design:**

Quasi-experimental study with interrupted time-series analysis.

**Setting:**

Non-teaching, urban, and community medical center within a multi-hospital health system.

**Patients:**

Adult inpatients with a beta-lactam allergy receiving antimicrobial therapy.

**Methods:**

The multidisciplinary CAMP team screened, interviewed, and collected allergy history details of adult inpatients with a beta-lactam allergy receiving antimicrobial therapy starting January 4, 2021. Patients were stratified as high, moderate, or low risk of IgE-mediated allergy and referred to an allergist for skin testing or drug challenge. The EMR was updated with interview details and drug challenge or skin test results. The primary endpoint was rate of complete allergy history documentation before (12/1/18–4/1/19) compared to after (1/4/21–5/1/21) program implementation. The secondary endpoint was days of inpatient beta-lactam therapy. Implementation logistics, de-labeling rate, and antimicrobial therapy changes were evaluated.

**Results:**

The program evaluated 392 individuals, with 184 and 208 patients comprising the pre- and post-intervention groups, respectively. The post-intervention period was associated with an increase of 19.8% in complete allergy histories (0.359 PPc; *R* ^2^ 0.26; *p* = 0.002) and 9.34 beta-lactam DOT per 1,000-days-present (1.106 PPc; *R* ^2^ 0.194; *p* = 0.009).

**Conclusion:**

Implementation of a comprehensive beta-lactam allergy management program was associated with higher rates of complete beta-lactam allergy history and beta-lactam use.

## Introduction

Hypersensitivity reactions are categorized into 4 types based on cell mediators involved in the immune reaction. Type I reactions are mediated by IgE antibodies and present with pruritus, flushing, angioedema, or bronchospasm within minutes to hours of allergen exposure.^1^ Medication allergies make up 5%–10% of adverse drug reactions in the United States, most of which are presumed to be type I, IgE-mediated reactions.^2^ Beta-lactam antibiotics comprise the most common drug allergy class in the U.S. with prevalence rates of 8%–20%, depending on the population evaluated.^3–9^ The U.S. Centers for Disease Control and Prevention suggest that less than 1% of these patients have an IgE-mediated reaction when evaluated by penicillin skin test (PST), with a negative predictive value greater than 97%.^10,11^ Additionally, approximately 80% of patients with IgE-mediated reactions lose sensitivity after 10 years, creating an opportunity to clarify allergies in these patients.^12^ In a pilot study at our institution, 10.2% of patient encounters in 2017 had a documented beta-lactam allergy, and merely 11.9% of those encounters had a reaction documented.^13^ Many of these allergies were suspected to be misclassified adverse drug reactions or intolerances.

The American Academy of Allergy, Asthma & Immunology’s Choose Wisely campaign recommends against overusing non-beta-lactam antibiotics in patients with a history of penicillin allergy without appropriate evaluation.^14^ The Infectious Diseases Society of America’s antimicrobial stewardship guidelines highlight detailed allergy history and PST as critical components of stewardship programs to improve first-line antibiotic use.^15–17^ Allergy misidentification and subsequent beta-lactam avoidance increases use of suboptimal, alternative antimicrobials. This worsens infection-related outcomes, increases patients’ length of hospital stay, potentiates antimicrobial resistance, and increases methicillin-resistant *Staphylocccus aureus*, vancomycin-resistant Enterococcus, and *Clostridioides difficile* infections.^18,19^

Limited data exists regarding characteristics, implementation logistics, and outcomes of allergy management programs in non-teaching, community hospitals.^20^ Much of the published literature describing successful programs includes medical resident and infectious disease (ID) fellow collaboration or are in states supporting pharmacist-administered PST. Our facility is a non-teaching, urban, community medical center within a multi-hospital health system. As a quality improvement initiative, the Beta-lactam Comprehensive Allergy Management Program (CAMP) was developed by a team of hospitalists, pharmacists, ID physicians, and an allergy and immunology physician. Program goals were to facilitate documentation of detailed medication allergy histories in the electronic medical record (EMR), increase targeted beta-lactam utilization, and educate patients and the clinical workforce on the impact of drug allergies on treatment. The study objective was to assess the rate of complete beta-lactam allergy histories and days of beta-lactam therapy before versus after CAMP implementation.

## Methods

This study was conducted at a hospital with an established PST program. However, program impact was limited by inconsistent workflow in candidate identification and testing processes. The team consisted primarily of 1 pharmacist and 1 allergist. This study evaluates impact of an expanded, multidisciplinary allergy management program implemented on January 4, 2021, to improve medication allergy documentation and increase use of targeted beta-lactams. A pharmacist reviewed charts and a daily EMR allergy report to identify adult inpatients with an EMR-documented beta-lactam allergy who were receiving antimicrobial therapy, irrespective of appropriateness. Patients were ineligible if the CAMP team was unable to obtain the patient’s allergy history (e.g. patient intubated, unable to contact family, etc.).

The CAMP team interviewed eligible patients to collect detailed medication allergy histories using a pharmacist-developed questionnaire (Table S1).^20,21^ In collaboration with an allergy and immunology physician, interviewed patients were stratified as high, moderate, or low risk of IgE-mediated allergy based on reaction history (Figure S1). Direct beta-lactam drug challenge was recommended for patients with low-risk histories. Moderate-risk patients were recommended to be further evaluated with PST. Beta-lactam avoidance was recommended in high-risk patients. The interviewer documented the allergy history questionnaire as an EMR progress note. The allergist removed the EMR allergy (de-label) and added the comment “Patient tested NEGATIVE via penicillin skin test/drug challenge by (practitioner name) on (date)” if patients tested negative via drug challenge or PST.

For de-labeled patients, the pharmacist and allergist collaborated with hospitalists or ID consultants to optimize antimicrobial therapy and increase use of beta-lactams, as appropriate. Patients were counseled on the results and provided with a wallet card with name, date, PST results, and allergist’s contact information. Additionally, a pharmacist communicated allergy results with patients’ outpatient physician and pharmacy before hospital discharge to update allergy documentation and ensure streamlined care.

Study analysis included adult inpatients with a beta-lactam allergy in the EMR and receiving any antimicrobial therapy. Pre- and post-intervention groups included patients admitted from December 1, 2018, to April 1, 2019, and January 4 to May 4, 2021, respectively. Study dates were selected to minimize the impact of the COVID-19 pandemic on program implementation by excluding the initial several months of the pandemic. The Institutional Review Board and Institutional Review Committee approved the study, with waiver of informed consent.

Primary endpoint was rate of complete allergy histories, defined as both medication and reaction documented in the EMR. Secondary endpoint was days of inpatient beta-lactam therapy (DOT) per 1,000-days-present in patients meeting inclusion criteria. Percentage of patients receiving a beta-lactam was evaluated and defined as receipt of a beta-lactam for at least 2 calendar days. Specific beta-lactam agents included in these outcomes are identified in Table S2. Study outcomes were assessed before versus after CAMP implementation.

Rate of complete allergy histories and beta-lactam DOT per 1,000-days-present were analyzed with interrupted time series using auto-regressive integrated moving average model (ARIMA). Each model evaluated the pre- versus post-intervention periods by including a binary dummy variable in the ARIMA model. The Breusch-Godfrey test and visual inspection of the plots determined autocorrelation and partial autocorrelation (Figure S2). These data were used to select the optimal model type (0,0,0), which was confirmed using Ljung-Box Q. Percentage point change was estimated for each study period using the ARIMA model. Statistical analysis was conducted using SPSS Statistics (Version 27). Hypothesis-generating outcomes are presented with descriptive statistics, including measures of central tendency and dispersion.

Not all patients meeting inclusion criteria were interviewed by the CAMP team in the post-intervention group due to COVID-19 infection control restrictions. Staff exposure risk was determined on a case-by-case basis. As such, a posthoc assessment was conducted to highlight the direct impact of program interventions by comparing outcomes within the post-intervention group between patients that were interviewed by a CAMP team member, referred to as the “CAMP cohort,” and those that did not have a CAMP team intervention, but still met inclusion criteria, referred to as the “non-CAMP cohort.”

## Results

### Patient population

**Figure 1.**
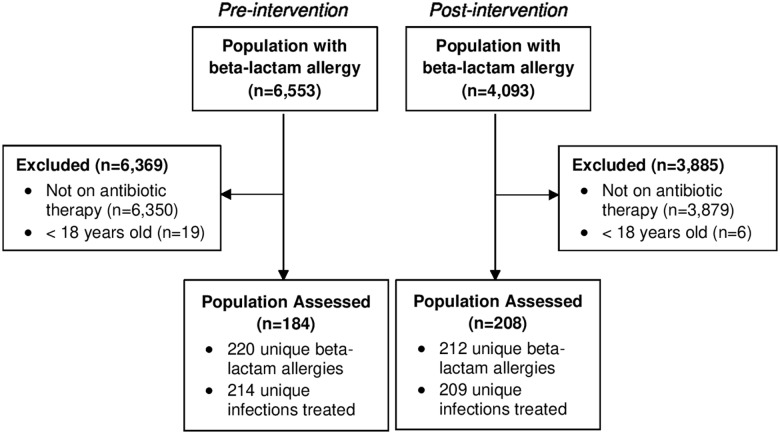
CONSORT diagram. *Note*: Patient enrollment and allocation.

Between December 1, 2018, and April 1, 2019, 6,553 patients had a beta-lactam allergy documented in the EMR. After excluding those without inpatient antimicrobial therapy or under 18 years old, 184 patients were included in the pre-intervention group. There were 220 distinct beta-lactam allergies identified and 214 infections treated in this group as patients may have had multiple drug allergens or infection indications documented. After program implementation, between January 4, 2021, and May 4, 2021, 4,093 patients had a beta-lactam allergy documented in the EMR. In the post-intervention group, 208 patients were included with 212 unique beta-lactam allergies identified and 209 infections treated (Figure 1).

Pre- and post-intervention groups had patients with mean age of 59.3 years (*p* = 0.99) and similar prevalence of female patients (73.3% vs. 74%; *p* = 0.92). Mean length of hospital stay was higher in the preintervention group (9.3 vs. 7.8 days-present; *p* = 0.56). Antimicrobial indications included urinary tract infection (24.8% vs. 20.1%; *p* = 0.25), pulmonary infection (21% vs. 15.8%; *p* = 0.16), skin and soft tissue infection (19.6% vs. 23.9%; *p* = 0.28), intra-abdominal infection (13.6% vs. 8.6%; *p* = 0.11), surgical prophylaxis (6.5% vs. 20.1%; *p* < 0.01), and other infection (14.5% vs. 11.5%; *p* = 0.36). Additional baseline characteristics are in Table 1.

**Table 1.** Baseline characteristics

Characteristic	Pre-intervention (*N* = 184)	Post-intervention (*N* = 208)	*P*-value
Age – year, mean (SD)	59.3 (21.6)	59.3 (19.1)	0.99
Female sex – no. (%)	137 (74.5)	154 (74.0)	0.92
Race – no. (%)			
White	61 (33.2)	76 (36.5)	0.48
Black	**60 (32.6)**	**49 (23.6)**	**0.05**
Asian Indian	2 (1.0)	0 (0.0)	0.23
Other	61 (33.2)	83 (39.9)	0.17
Days present – days, mean (SD)	9.3 (24.0)	7.8 (26.1)	0.56
Antibiotic indication – no. (%)	*n* = 214^a^	*n* = 209^a^	
Urinary tract	53 (24.8)	42 (20.1)	0.25
Pulmonary	45 (21.0)	33 (15.8)	0.16
Skin and soft tissue	42 (19.6)	50 (23.9)	0.28
Intra-abdominal	29 (13.6)	18 (8.6)	0.11
Surgical prophylaxis	**14 (6.5)**	**42 (20.1)**	**<0.01**
Other	31 (14.5)	24 (11.5)	0.36

a Some patients were treated for multiple infections; therefore, the total number of infections is greater than total number of patients.

### Primary and secondary outcomes

**Figure 2.**
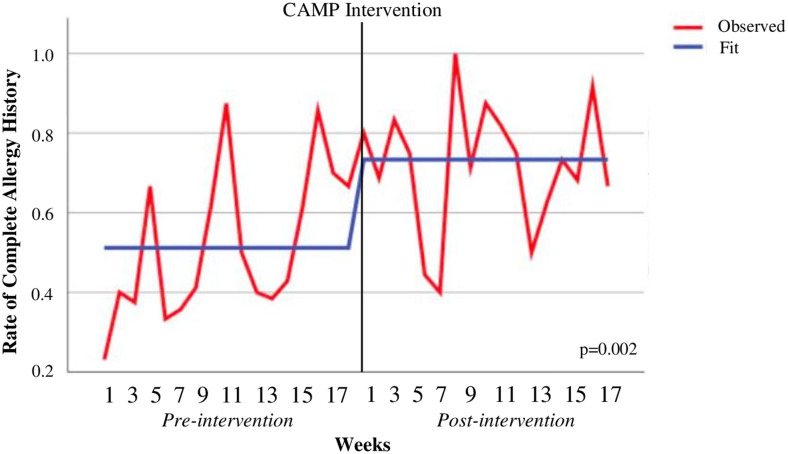
Complete allergy history. *Note*: The interrupted time-series analysis using auto-regressive integrated moving average model model (0,0,0) is shown in this figure.

**Figure 3.**
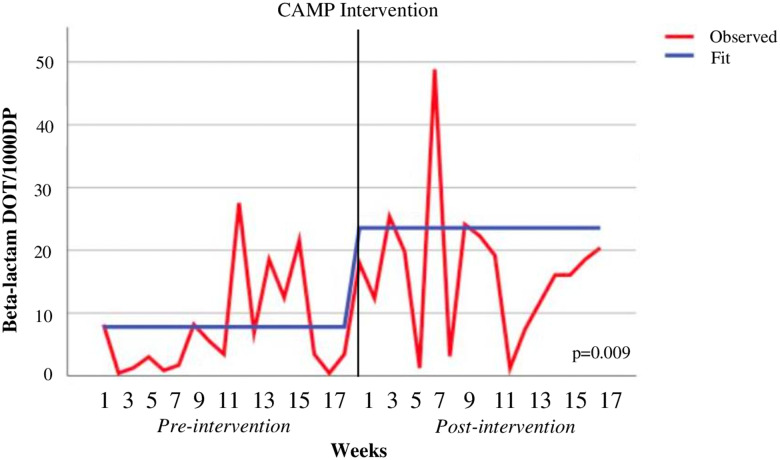
Beta-lactam days of therapy per 1,000-days-present. *Note*: The interrupted time-series analysis using auto-regressive integrated moving average model model (0,0,0) is shown in this figure; *DOT/1000DP, days of therapy per 1000-days-present.*

Primary and secondary outcomes were evaluated as an interrupted time-series analysis using average of weekly complete allergy history rates and beta-lactam DOT per 1,000-days-present, respectively. The average of weekly complete allergy histories increased by 19.8% from 51.9% [42.2–61.6] in the pre-intervention group to 71.7% [63.5–80.0] in the post-intervention group (*R* ^2^ = 0.26; 0.359 estimate (PPc); *p* = 0.002) (Table 2, Figure 2). The average of weekly beta-lactam DOT per 1,000-days-present increased by 9.34 DOT per 1,000-days-present from 7.47 [3.33–11.6] in the pre-intervention group to 16.81 [11.03–22.59] in the post-intervention group (*R* ^2^ = 0.194; 1.106 estimate (PPc); *p* = 0.009) (Table 2, Figure 3).

**Table 2.** Interrupted time series model

Outcome	ARIMA (p,d,q)	Stationary *R* ^2^	Estimate (PPc)	*t*-value	*p*-value
Complete Allergy History	0,0,0	0.260	0.359	3.349	0.002
Beta-lactam DOT per 1,000-days-present	0,0,0	0.194	1.106	2.778	0.009

Note. Model components were pre- versus post-intervention.

### Exploratory outcomes

Aggregate beta-lactam allergy histories were complete with allergen and reaction identified in a total of 108 of 220 documented allergies (49.1%) in the pre-intervention group compared to 151 of 212 (71.2%) post-intervention. Total beta-lactam DOT increased from 126.9 to 285.7 DOT per 1,000-days-present in all included patients on antibiotics with a documented beta-lactam allergy. Patients receiving any beta-lactam increased by 13.3% [26.6% (49/184) vs. 39.9% (83/208)]. In these individuals treated with beta-lactam, median beta-lactam DOT also increased (Table 3). DOT per 1,000-days-present increased for penicillins (6.5 vs. 43.3), cephalosporins (74.4 vs. 181.8), and carbapenems (46.0 vs. 60.6) after program implementation.

**Table 3.** Patients on beta-lactam therapy

	Pre-intervention		Post-intervention	
	Patients who received Beta-lactam (***n*** = 49)	Total Population (*N* = 184)	Patients who received Beta-lactam (*n* = 83)	Total Population (*N* = 208)
Mean beta-lactam DOT/1000DP (SD)	2.64 (3.54)	0.69 (2.14)	3.84 (3.90)	1.51 (3.08)
Median beta-lactam DOT/1000DP (IQR)	0.86 (0.43–3.55)	0 (0–0.43)	2.47 (1.24–5.57)	0 (0–1.86)

Note. *DOT/1000DP*, days of therapy per 1,000-days-present.

### Posthoc assessment

**Figure 4.**
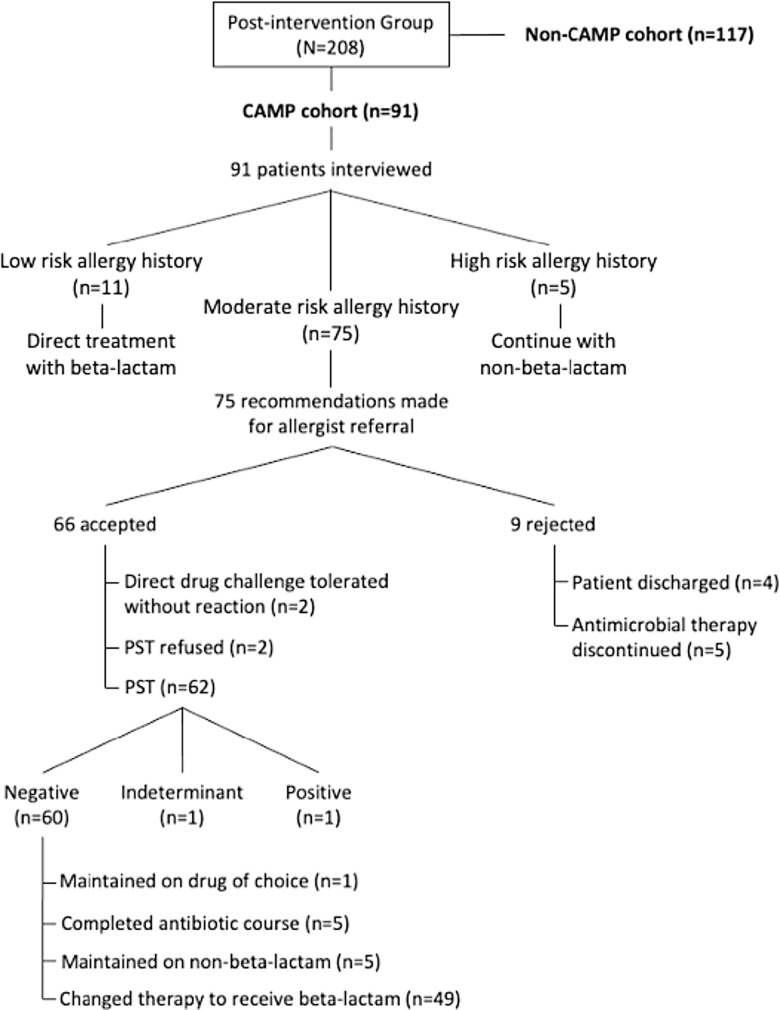
Posthoc assessment of Comprehensive Allergy Management Program (CAMP) interventions. *Note*: The study’s post-intervention group was further divided posthoc into those patients with targeted intervention (CAMP cohort) and those without (non-CAMP cohort). Specific interventions in the CAMP cohort are depicted in this flow diagram.

Comprehensive Allergy Management Program team members conducted 91 patient interviews, referred to as the “CAMP-cohort.” The median interview time was 10 minutes (IQR 5–15). Patients interviewed by a CAMP team member had a 100% rate of complete allergy histories. Seventy-five recommendations were made for allergist referral and 66 were accepted (88%). Rejections were due to the treatment team’s decision to discontinue antibiotics (*n* = 5) or discharge on interview day (*n* = 4). Sixty-two patients underwent PST, 2 received beta-lactam drug challenge, and 2 refused PST. Both challenged patients tolerated their respective beta-lactam antibiotics without reaction. Sixty patients tested negative upon PST, 1 patient tested positive, and 1 patient had an indeterminate reading due to antihistamine administration 48 hours prior to PST. A total of 62 patients were de-labeled due to negative skin test or drug challenge (68.1% of the “CAMP-cohort”). No patient had allergic symptoms after de-labeling by hospital discharge. Fifty-four patients were eligible for antibiotic change to a beta-lactam after negative PST and 49 therapies were modified (90.7%). The remaining 5 patients were maintained on non-beta-lactam agents per prescriber discretion. There were 6 patients not eligible for therapy change due to antibiotic course completion (*n* = 5) or already receiving a first-line antibiotic (*n* = 1). Program flow diagram is depicted in Figure 4.

## Discussion

The CAMP was developed as an antimicrobial stewardship initiative at a non-teaching, community medical center by a small group of pharmacists, physicians, and 1 allergy specialist. Despite limited personnel and pandemic restrictions, statistically significant increases were seen in complete allergy histories and beta-lactam DOT after program implementation. Several studies have established the clinical impact of appropriate allergy reporting, subsequent increases in beta-lactam use, and the tolerability of beta-lactams with disparate R1-group side chains than that of drug allergen.^22–24^ Griffith and colleagues developed a penicillin allergy assessment and skin testing service at a tertiary care center where the rate of reconciled penicillin allergies improved from 11% to 75% after program implementation (*p* < 0.0001). Penicillin class antibiotic use increased from 0% to 37% after PST (*p* < 0.05).^20^ To our knowledge, this is the first study highlighting operational characteristics, implementation logistics, and outcomes of a comprehensive allergy management program in a facility without medical residents or ID fellow presence and in a state where pharmacists are not licensed to conduct PSTs.

Program interventions were associated with an increase in complete allergy histories, overall beta-lactam DOT, and number of patients receiving any beta-lactams. The increase in beta-lactam utilization was attributable to complete allergy histories, PST, and de-labeling. Penicillin class utilization rose numerically post-intervention (6.5 vs. 43.3 DOT per 1,000-days-present). Cephalosporin use increased (74.4 vs. 181.8 DOT per 1,000-days-present) as ceftriaxone and cefepime are recommended empiric drugs of choice for common infections based on institution-specific antibiogram and treatment algorithms. Carbapenem utilization remained unchanged (46 vs. 60.6 DOT per 1,000-days-present) likely due to low cross-reactivity with penicillin allergies leading to prescriber comfort at baseline with carbapenem initiation without PST. Study results inspired greater confidence in allergy evaluation and risk stratification, encouraging providers to prescribe these agents to low-risk patients even without PST. This manuscript confirms, combines, and expands upon various facets of other studies including pragmatic outcomes from a multidisciplinary approach to allergy history documentation and skin testing.

### Impact of appropriate documentation

Prevalence studies suggest that complete allergy history documentation is uncommon, with incomplete or absent reactions in 50%–80% of patients.^5,7,8^ Mann and colleagues interviewed 175 patients and found that 133 (76%) required a change to their allergy profile for reaction inclusion or allergen specification.^21^ A pilot study at our institution in 2017 showed 13,380 beta-lactam allergies documented; however, only 1,592 (11.9%) had a reaction documented.^13^ Our institution has since mandated allergy reaction documentation; however, this study showed that aggregate, complete history documentation of beta-lactam allergies was only 49.1%, with the remaining 50.9% having reaction listed as “unknown.” Comprehensive Allergy Management Program implementation was associated with an aggregate 22.1% increase in complete beta-lactam allergy histories (*R* ^2^ = 0.26). Since the CAMP team only interviewed 91 of 208 eligible patients in the post-intervention group (43.8%), this effect size may be underestimated. This improvement was similar to Griffith and colleagues’ observed outcomes.^20^ Complete documentation of specific beta-lactams and reactions allows for greater confidence in beta-lactam utilization despite a listed allergy.^9^

### Antibiotic days of therapy

The comprehensive allergy management program was associated with enhanced utilization of beta-lactam therapy (*R* ^2^ = 0.194). Our study assessed antimicrobial therapy utilization rather than appropriateness. However, previous publications have shown an increase in appropriateness secondary to PST.^20^ Mann and colleagues also illustrated that complete allergy histories can elevate antimicrobial therapy appropriateness, with 42 of 135 (31.1%) patients transitioned to an appropriate non-carbapenem beta-lactam antibiotic with a penicillin allergy interview protocol.^25^

### Clinicians’ role

Multidisciplinary collaboration was instrumental in program success. Hospitalists and ID physicians recognized appropriate candidates, reconciled medication allergy histories, and transitioned patients to beta-lactam therapy after negative PST. The allergist conducted the skin testing and interpreted results. Pharmacists played a pivotal role by conducting patient interviews, assisting with drug challenges and PST, and optimizing medication therapy in collaboration with physicians. Pharmacists also provided in-services to providers, nurses, and pharmacy personnel regarding the program’s antimicrobial stewardship initiatives, the medication allergy history interview tool, medication allergy assessment algorithm, and drug challenge and PST protocols. Reference materials provided to the clinical workforce are included in the Supplementary Material. The allergist and pharmacists counseled patients on PST results and ensured de-labeling of allergies in the EMR after negative test result. Our study highlights the impact of various healthcare professionals and the collective success of allergy management in a non-academic or tertiary care environment.

### Future directions

The CAMP initiative was well received by prescribers, nurses, and pharmacists. Prescribers’ comfort with CAMP intervention increased as the program evolved and led to multidisciplinary collaboration in allergy clarifications and antimicrobial stewardship. At our institution, nurses are responsible for allergy documentation. Increasing nursing involvement in this initiative can enhance the number of patients with complete documentation and subsequently increase drug challenge and PST referrals. Additionally, increasing the number of clinicians involved in drug challenges and PST will expand institutional capacity for allergy services. Hospitalists have a significant impact with a vast range and volume of patients under their care. Program growth can be supported by providing hospitalists with PST certification programs.

The American Society of Health-System Pharmacists advocates for pharmacist involvement in clarifying beta-lactam allergies by assisting with documentation and de-labeling of beta-lactam allergies, PST, and direct antibiotic challenges in appropriate candidates.^26^ However, current state regulations prevent pharmacists from conducting drug challenges and PST independently in 13 states. With provider status or collaborative practice agreements, pharmacists could expand these services within their scope of practice.^27^

Overall, this program sets the framework for complete documentation of all drug allergens, not only beta-lactams, impacting clinical and patient outcomes. The authors plan on conducting a subsequent analysis specifically on patients who have undergone PST at our institution to evaluate beta-lactam versus non-beta-lactam DOT as this study’s primary aim was to highlight CAMP team’s impact on complete allergy history documentation.

### Limitations

Given the small sample size at a single institution, results may not be generalizable. However, not all eligible patients were interviewed by the CAMP team due to COVID-19 exposure risks, limited personnel availability, or short hospital stays. Therefore, true effect size may be underestimated. Patients interviewed by a CAMP team member had 100% rate of complete allergy histories. Due to these restrictions, patients targeted for interview may have been inadvertently prioritized by their severity of illness, introducing the risk of channeling bias. Allergy reaction histories were self-reported, which may introduce recall bias. Lastly, all providers may not be aware of or trust allergy de-labeling results, impacting antibiotic prescribing trends.

## Conclusions

The comprehensive beta-lactam allergy management program was associated with a higher rate of complete beta-lactam allergy histories in the EMR and use of beta-lactam therapies.

## Supporting information

Vyas et al. supplementary material Vyas et al. supplementary material

## Supplementary material

For supplementary material accompanying this paper visit http://doi.org/10.1017/ash.2023.461. click here to view supplementary material
