# Supplementary material for: Beta-lactam comprehensive allergy management program in a community medical center
Source: Antimicrob Steward Healthc Epidemiol. 2023 Oct 27;3(1):e189. doi: 10.1017/ash.2023.461 (PMC10654959; doi:10.1017/ash.2023.461)
Supplement: Vyas et al. supplementary material [file S2732494X23004618sup001.pdf]

Supplementary Appendix

Beta-lactam Comprehensive Allergy Management Program in a Community Medical Center

Lakhini Vyas, PharmD, BCPS; Karan Raja, PharmD, BCPS, BCIDP, AAHIVP; Susan Morrison, MD; Donald Beggs, MD; Mark S. Attalla, PharmD, MBA; Mitesh Patel, PharmD, BCCCP; Mona Philips, RPh, MAS

Table S1. Medication Allergy History Interview Tool

Patient Questionnaire[20-21]
What medication or medication class was the allergy to?
What route of administration was the medication? (e.g. IV, PO, IM, SC)
What were you taking the medication for? What else were you sick with at the time? (e.g. viral infection, bacterial infection, etc.)
How many doses of the medication did you take before the reaction?
What was the reaction? If rash, do you have a photo?
How long ago did the reaction occur?
How long after taking the antibiotic did the reaction occur?
How and when did the reaction resolve?
Were you hospitalized and/or given medication to treat the reaction?
Were you taking any other medications at the time of reaction?
What antibiotics do you normally take when you are sick? (e.g. penicillin, amoxicillin, amoxicillin-clavulanate, ceftriaxone, cefepime, doxycycline, ciprofloxacin, etc.)
Do you have a family history of beta-lactam allergy without a personal history of the allergy?
Were you ever referred to an allergy specialist? If yes, when and what was the result?
Other comments:

Table S2. Beta-lactam Class Antimicrobials

**Penicillins**		
Penicillin G	Penicillin V	Oxacillin
Nafcillin	Dicloxacillin	Amoxicillin
Ampicillin	Amoxicillin-clavulanic acid	Ampicillin-sulbactam
Piperacillin-tazobactam		
**Cephalosporins**		
Cefazolin	Cephalexin	Cefadroxil
Cefuroxime	Cefoxitin	Cefaclor
Cefprozil	Cefotaxime	Ceftriaxone
Cefpodoxime	Cefixime	Cefdinir
Ceftazidime	Ceftazidime-avibactam	Cefepime
Ceftolozone-tazobactam	Ceftaroline	
**Carbapenems**		
Imipenem-cilastin	Meropenem	Ertapenem

Legend: Study definition of “beta-lactams” consisted of all potential beta-lactam class antimicrobials, including those that may not have been on the institution’s formulary. Therefore, some agents listed above were not used in the study population.

Figure S1: Medication Allergy Assessment Algorithm

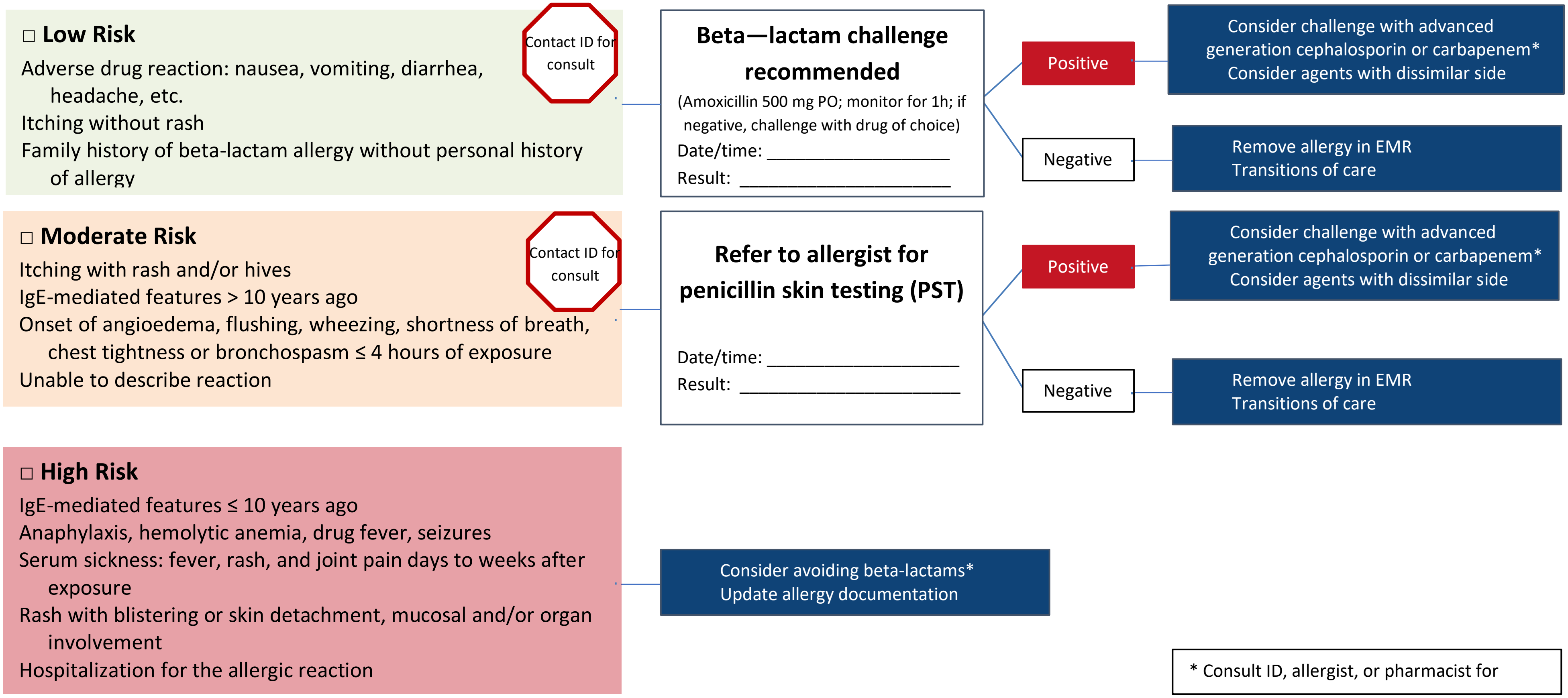

Legend: Medication allergy assessment algorithm to stratify risk of IgE-mediated reaction and provide recommendations to optimize antimicrobial therapy

Figure S2: ARIMA Sequence Charts

(a) Primary Endpoint – Complete Allergy Histories

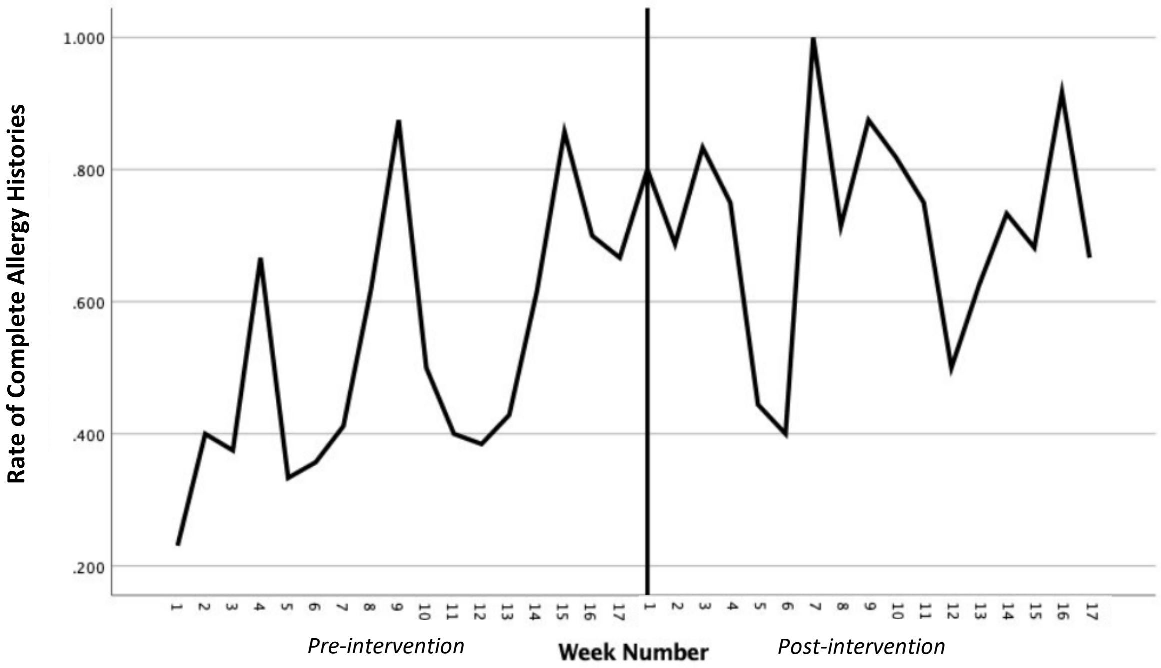

(b) Secondary Endpoint – Beta-lactam days of therapy per 1000-days-present

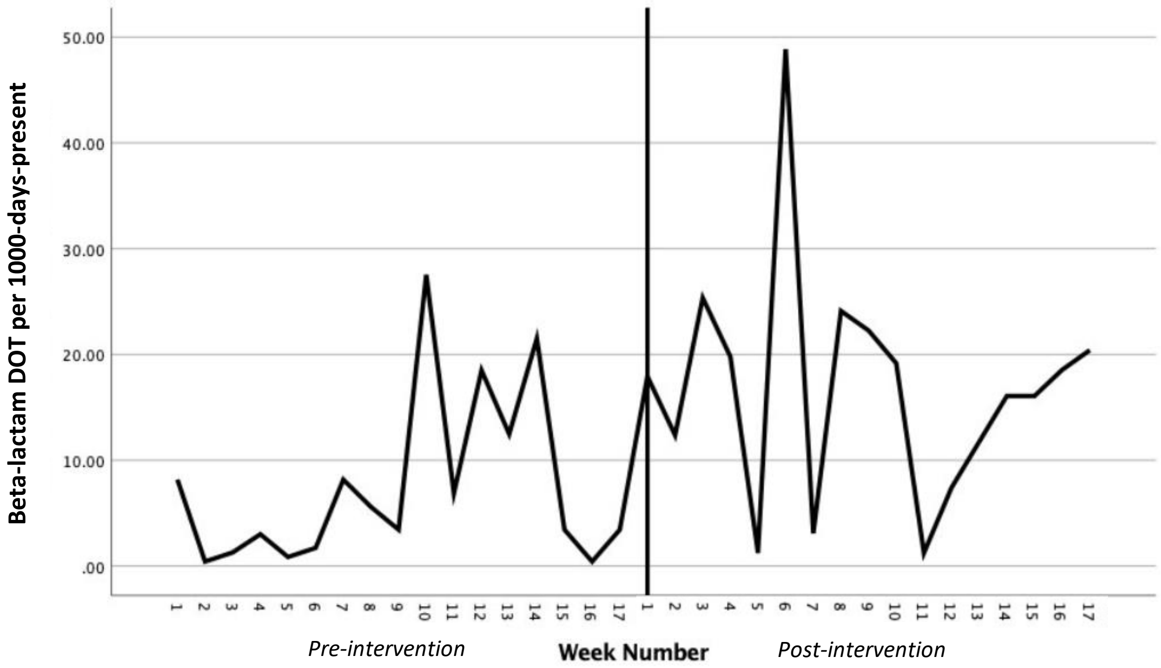

Legend: Sequence charts identifying trends and evaluating autocorrelation and partial autocorrelation
